# Potential Cost-Effectiveness of an Influenza Vaccination Program Offering Microneedle Patch for Vaccine Delivery in Children

**DOI:** 10.1371/journal.pone.0169030

**Published:** 2016-12-22

**Authors:** Carlos Wong, Minghuan Jiang, Joyce H. S. You

**Affiliations:** School of Pharmacy, Faculty of Medicine, The Chinese University of Hong Kong, Shatin, N.T, Hong Kong; Instituto Butantan, BRAZIL

## Abstract

**Objective:**

The influenza vaccine coverage rate of children is low in Hong Kong. Microneedle patches (MNPs) is a technology under development for painless delivery of vaccines. This study aimed to examine the potential clinical outcomes and direct medical costs of an influenza program offering MNP vaccine to children who have declined intramuscular (IM) vaccine in Hong Kong.

**Methods:**

A decision model was designed to compare potential outcomes between IM vaccine program and a program offering MNP vaccine to those declined IM vaccine (IM/MNP program) in a hypothetical cohort of children over one-year time horizon. The model outcomes included direct medical cost, influenza infection rate, mortality rate, and quality-adjusted life-years (QALYs) loss. Model inputs were retrieved from published literature. Sensitivity analyses were performed to examine the robustness of model results.

**Results:**

In base-case analysis, IM/MNP program was more costly per child (USD19.13 versus USD13.69; USD1 = HKD7.8) with lower influenza infection rate (98.9 versus 124.8 per 1,000 children), hospitalization rate (0.83 versus 1.05 per 1,000 children) and influenza-related mortality rate (0.00042 versus 0.00052 per 1,000 children) when compared to IM program. The incremental cost per QALY saved (ICER) of IM/MNP program versus IM program was 27,200 USD/QALY. Using gross domestic product (GDP) per capita of Hong Kong (USD40,594) as threshold of willingness-to-pay (WTP) per QALY, one-way sensitivity analysis found ICER of IM/MNP to exceed WTP when duration of illness in outpatient setting was <5.7 days or cost per MNP vaccine was >1.39-time of IM vaccine cost. In 10,000 Monte Carlo simulations, IM/MNP program was the preferred option in 57.28% and 91.68% of the time, using 1x and 3x GDP per capita as WTP threshold, respectively.

**Conclusion:**

Acceptance of IM/MNP program as the preferred program was subject to the WTP threshold, duration of illness in outpatient settings, and cost of MNP vaccine.

## Introduction

In Hong Kong, the hospitalization rate for influenza in children aged 0–4 years has increased from 0.73 per 10,000 in 2013 to 2.49 per 10,000 in 2016 during peak influenza season, suggesting that the influenza activity has continued to increase annually [1]. The most frequent influenza-associated complication in children aged 0–4 years was acute otitis media and acute respiratory infections [2]. The mortality rate of influenza infection in children aged 5–14 years during 2009 pandemic H1N1 influenza in Hong Kong was approximately 0.4 per 100,000 infections [3].

The Childhood Influenza Vaccination Subsidy Scheme offered by the Hong Kong Department of Health subsidizes HKD160 (USD1 = HKD7.8) per dose of seasonal influenza vaccination in children between 6 months and 6 years of age [4]. Currently, intramuscular (IM) formulation of quadrivalent influenza vaccine is administered to children in public healthcare facilities. Despite the government subsidy, the number of influenza vaccines provided by government to children in 2016 was lower than the number in 2015 by 25%, and the acceptance rate of influenza vaccination in children is low (28.4%) [5]. A community-based survey on immunization in Hong Kong school-age children showed that fear of needle jab was a major cause for declining vaccination [6].

Intradermal injection is an alternative route of administration for influenza vaccine. Intradermal influenza vaccine targeted at the epidermal Langerhans cells stimulates a greater immune response in the recipients than IM injection, and therefore achieves a dose sparing effect [7]. Microneedle patches (MNPs) with array of coated solid-microneedle or dissolving microneedles is a technology under development for intradermal delivery of vaccines. The microneedle technology offers a number of advantages for vaccine delivery: Avoidance of hypodermic needle-related phobia and risk; painless route of administration, simplification of storage, distribution, and disposal of vaccines, and potential self-administration [8]. A recent economic model assessed the potential impact of adding a vaccine product delivered by MNPs to the US influenza vaccine market [9]. It was reported that the MNP vaccine would be cost-effective or even cost-saving with QALYs saved when it was administered by healthcare providers. An influenza vaccination program offering needleless formulation to elderly who had declined IM formulation of vaccine was found to be highly cost-effective [10]. Offering MNP vaccine to children who have declined IM vaccine would potentially improve the acceptance of vaccination and cost-effectiveness of vaccination program from the perspective of healthcare provider and administrator. In this study, we examined the potential clinical and economic outcomes of an influenza program offering MNP vaccine to children who have declined the IM vaccine in Hong Kong.

## Methods

### Decision-analytic model

**Fig 1:**
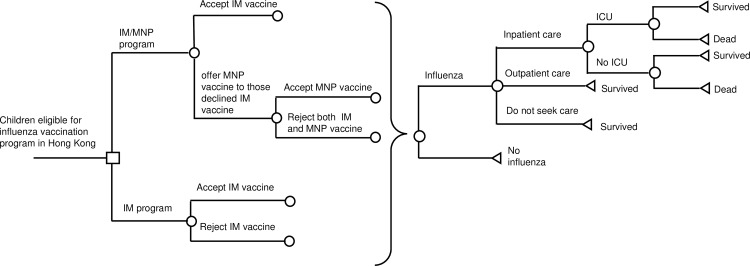
Simplified decision-analytic model. IM: intramuscular; MNP: microneedle patch; ICU: intensive care unit.

A decision-analytic model (Fig 1) was designed to compare the potential difference in outcomes between the current influenza program (IM program) and a program offering MNP vaccine to children who declined IM vaccine (IM/MNP program), in a hypothetical cohort of children aged 6 months to 6 years (all are eligible for the Childhood Influenza Vaccination Subsidy Scheme in Hong Kong). Children with contraindications to influenza vaccine were excluded from the present model. The time horizon for the analysis was one year. The outcomes of the decision model included direct medical cost, influenza infection rate, influenza-related mortality rate, and influenza-related quality-adjusted life-years (QALYs) loss.

In IM program, children would either accept IM vaccine or remain unvaccinated. In IM/MNP program, children would be first offered IM vaccine. MNP vaccine would be further offered to those who declined IM vaccine and they might accept or decline MNP vaccine. Vaccinated children might experience common adverse events of influenza vaccine including injection site reaction, fever and headache. All children in the model might be infected by influenza whether they were vaccinated or not. Infected children might be cared by self-treatment, outpatient or inpatient treatment. Children with inpatient care might survive or die, with or without being admitted to the pediatric intensive care unit (ICU).

### Clinical inputs

All model inputs were listed in Table 1. We conducted literature search on MEDLINE over the period of 1998–2016 using the keywords “influenza”, “influenza vaccine”, “vaccine effectiveness”, “vaccine coverage”, “vaccine acceptance”, “intramuscular”, “microneedle patch”, “influenza severity”, “children”, and “utility score”. The data retrieved for model inputs were screened for relevance of present model. Clinical findings such as influenza vaccine effectiveness and adverse events have high transferability between countries, and data reported in local and non-local population were both considered acceptable model inputs. Healthcare utilization has low transferability, and published data in local population were preferred for the present model. If a variable was reported in multiple studies, the weighted average was used as the base-case value, and the highest and lowest values formed the range for sensitivity analysis.

**Table 1:** Model Inputs.

	Base-case value	Range	Distribution type	Reference
**Clinical Inputs**				
Acceptance rate of IM influenza vaccine	0.284	0.227–0.341	Triangular	[5]
Increment of vaccine acceptance rate with MNP versus IM vaccine	1.33	1.03–1.641	Triangular	[12]
Probability of IM vaccine-related adverse events	0.0612	0.0490–0.0734	Beta	[13]
Relative risk of adverse events of MNP versus IM vaccine	1	0.8–1.2	Triangular	Assumption
Vaccine effectiveness	0.63	0.52–0.72	Triangular	[15]
Influenza infection rate in unvaccinated children	0.152	0.114–0.189	Triangular	[16]
Proportion of high-risk children	0.052	0.0416–0.0624	Triangular	[17]
Probability of outpatient visit in non-high-risk children	0.455	0.357–0.553	Gamma	[17]
Probability of outpatient visit in high-risk children	0.91	0.66–1	Gamma	[17]
Hospitalization rate per influenza infection	0.0084	0.0076–0.0097	Triangular	[3]
ICU admission rate during hospitalization	0.0094	0.0075–0.011	Triangular	[3]
Mortality rate of influenza infection	0.0005	0.0004–0.0006	Triangular	[3]
**Utility Inputs**				
Age of children (years)	4	0.5–6	Triangular	[11]
Utility score of children aged 2–6 years	1	-	-	[18]
Utility loss of vaccine-related adverse events	0.05	0.01–0.10	Triangular	[19]
Utility score of self-treated influenza	0.725	0.580–0.870	Triangular	[19]
Utility score of outpatient treatment	0.60	0.49–0.81	Triangular	[20]
Utility score of hospitalization	0.5	0.4–0.6	Triangular	[20]
Utility score of ICU care	0.38	0.304–0.456	Triangular	[20]
**Cost Inputs (USD)**				
IM vaccine (per dose)	16.7	-	Triangular	Local price
Incremental cost factor of MNP versus IM vaccine	1	1–2	-	Assumption
Percentage of children requiring 2 doses of vaccine	0.5	0–1	Triangular	Assumption
Treatment of vaccine adverse events	1.41	1.13–1.69	Triangular	OTC price
Self-treated influenza	5.92	4.74–7.11	Triangular	OTC price
Outpatient clinic visit (per visit)	49.4	-	-	[24]
Hospitalization in general pediatric ward (per day)	600	-	-	[24]
Hospitalization in pediatric ICU (per day)	2950	-	-	[24]
Duration of vaccine adverse events (days)	2	1–3	Uniform	Assumption
Duration of illness for outpatient care/self-treated influenza (days)	8.4	2.7–14.1	Gamma	[21]
Length of hospitalization for influenza (days)	4.94	1–6	Triangular	[22]
Number of clinic visit	1	1–2	Uniform	Assumption
IM: Intramuscular; MNP: microneedle patch; ICU: Intensive care unit; OTC: Over the counter				

The age of Hong Kong children (mode 4 years; range: 0.5–6 years) eligible for The Childhood Influenza Vaccination Subsidy Scheme was estimated from the Hong Kong 2016 population by age group [11]. The acceptance rate of IM vaccine (28.4%) was retrieved from findings of a community-based survey conducted by the Department of Health on influenza vaccine coverage in children aged 6 months to 5 years during 2012/13 season in Hong Kong [5]. A randomized, repeated measure study on usability and acceptability of MNPs for influenza vaccination in 91 healthy volunteers reported increased willingness to be vaccinated with MNP by 1.33-fold (range 1.03–1.64), comparing with non-MNP vaccine acceptance in the US [12]. The adverse event rate of IM vaccine (6.12%) was retrieved from a randomized clinical trial (N = 4,348) that evaluated the immunogenicity and adverse events of inactivated quadrivalent influenza vaccine in children [13]. A meta-analysis (6 clinical studies for 673 adult subjects) reporting no significant differences in the occurrence of adverse events with intradermal and IM influenza vaccines [14]. We therefore assumed the relative risk of adverse event with MNP vaccine to be similar to IM vaccine in present model (odds ratio = 1) and examined the assumption over a range of 0.8–1.2. The adjusted vaccine effectiveness for 2015–2016 season among children reported by the Centers for Disease Control and Prevention (CDC) was 63% (95% CI 52%-72%) [15]. The natural attack rate of influenza in unvaccinated children was reported by a meta-regression analysis of 34 randomized clinical trials to be 15.2% (95% CI 11.4%-18.9%) [16] and it was adopted as the model input for influenza infection rate in unvaccinated children. The infection rate of vaccinated children was calculated by:

Infection rate of unvaccinated children * (1—vaccine effectiveness).

The proportion of high-risk children (5.2%), and the probabilities of non-high-risk and high-risk children seeking outpatient care (45.5% and 91%, respectively) were derived from a study using probabilistic model and epidemiological data to measure the burden of seasonal influenza [17]. The hospitalization rate per infection (0.84%; 0.76%-0.97%); ICU admission rate per hospitalized case (0.94%; 0.75%-1.1%), and mortality rate (0.05%; 0.04%-0.06%) were retrieved from the findings of a local study assessing the infection influence of pandemic H1N1 influenza in children in 2009 [3].

### Utility and costs inputs

The QALYs loss of each child was the summation of QALYs loss for: vaccine-related adverse events, self-treated influenza, outpatient care, hospitalization without ICU admission, ICU care, and death. The QALYs loss of each event (except for death) was calculated by multiplying the loss of utility (utility scorechildren−utility score_event_) and time-spent in the event. Age-specific utility score was retrieved from a population-based quality of life validation analysis [18]. The utility score of each health state was derived from health-related life quality estimates and published economic studies on influenza testing or vaccine program [19,20]. The duration of vaccine adverse events was assumed to be 2 days (range 1–3 days) The time-spent in outpatient care (and self-treatment) and hospitalization were the duration of illness and length of hospital stay, correspondingly [21,22]. Time loss for death was retrieved from the age-specific life table reported by Hong Kong Census and Statistics Department [11]. QALYs loss for death was discounted by an annual rate of 3% to year 2016.

The cost analysis was conducted from the perspective of Hong Kong healthcare provider and included direct medical cost items at year 2016 costs. Direct medical cost included costs of vaccine, treatment of vaccine-related adverse events and treatment of influenza (self-care, outpatient care and inpatient care). In the present model, the cost of both IM and MNP vaccines were subsided by the public vaccination subsidy program. The cost per IM vaccine administrated was USD16.7 in Hong Kong and the potential cost per MNP vaccine administered was assumed to be the same as IM vaccine cost, and examined over a range of 1-2x IM vaccine cost. Children who accepted vaccination might receive 1–2 doses. Two doses were required for those who have never been vaccinated against influenza or with unknown influenza vaccination history [23]. We assumed 50% (range 0–100%) of children who accepted vaccination required 2 doses of influenza vaccine. The costs of vaccine-related adverse events and self-treatment of influenza infection were estimated by the costs of over-the-counter medications for fever, pain, cold, or influenza symptoms. Infected children were assumed to have one time clinical visit for outpatient care (range: 1–2 visits). The cost per clinic visit and daily cost of inpatient hospitalization were retrieved from the charges for services listed by the Hospital Authority (the largest public healthcare provider in Hong Kong), assuming the charge to represent the cost of service with no additional profits in the public healthcare setting [24].

### Base-case analysis and sensitivity analysis

Base-case and sensitivity analyses were performed by using TreeAge Pro 2015 software (TreeAge Software Inc., Williamstown, MA) and Microsoft Excel 2013 (Microsoft Corporation, Redmond, WA, USA). In the base-case analysis, the expected values of four model outputs (direct medical cost, influenza infection rate, influenza-related mortality rate, and QALYs loss) were calculated using the base-case values of model parameters. If the IM/MNP program was more effective in saving QALY at higher cost, the incremental cost-effectiveness ratio (ICER) of IM/MNP program would be calculated: (Cost_IM/MNP_−Cost_IM_)/(QALY loss_IM_−QALY loss_IM/MNP_). The World Health Organization’s recommendation on region-specific cost-effectiveness threshold of willingness-of-pay (WTP) suggested that a strategy with ICER less than 1x gross domestic product (GDP) per capita to be highly cost-effective, and less than 3x GDP per capita to be cost-effective [25]. The GDP per capita in Hong Kong was USD40,594 (USD1 = HKD7.8) in 2015 [26], and USD40,594 (1x GDP per capita) and USD121,782 (3x GPD per capita) were adopted as the WTP thresholds for highly cost-effective and cost-effective, respectively, in the present analysis.

Sensitivity analysis was performed to examine the robustness of base-case results. One-way sensitivity analysis was performed with all parameter ranges (95% CI or ±20% of base-case values) to identify the influential factors on base-case analysis. The vaccine effectiveness and baseline influenza infection rate were two influenza surveillance parameters frequently reported as key predictors on effectiveness of vaccination programs [9,10,19,27]. Prior economic analyses showed that high-cost vaccine was only cost-effective when both vaccine effectiveness and baseline infection rate were high [10]. The MNP technology is not yet fully licensed for production, thus the vaccine cost is not available. A three-way sensitivity analysis was therefore conducted to examine the interaction between influenza infection rate in unvaccinated children, vaccine effectiveness and three potential cost levels of MNP vaccine (1x, 1.25x, and 1.5x relative to IM vaccine cost) on the cost-effectiveness of IM/MNP program. To assess the impact of all variables simultaneously, probabilistic sensitivity analysis was performed for 10,000 Monte Carlo simulations by randomly drawing each of model inputs with a specific probability distribution (as indicated on Table 1). The probability of each program to be the preferred option was determined over a wide range of 1–3x GDP per capita as WTP threshold in an acceptability curve.

## Results

### Base-case analysis

The base-case results were shown in Table 2. The IM/MNP program was more costly with lower influenza infection rate, hospitalization rate and influenza-related mortality rate, and saved QALY when compared to IM program. The ICER of IM/MNP program versus IM program was 27,200 USD/QALY saved, and it was lower than 1x GPD per capital (40,594 USD/QALY saved).

**Table 2:** Base-case results of influenza-related direct medical cost, infection rate, mortality rate and QALY loss.

Strategy	Cost^a^ (USD)	Influenza infection rate^b^	Hospitalization rate^b^	Influenza-associated morality rate^b^	QALY loss^a^	ICER (USD/QALY saved)
IM program	13.69	124.8	1.05	0.00052	0.00098	-
IM/MNP program	19.13	98.9	0.83	0.00042	0.00078	27,200

a Cost and QALY loss per child (in year 2016) to whom vaccination program was offered

b Event rate per 1000 children to whom vaccination program was offered

IM: intramuscular; MNP: microneedle patch; QALY: quality-adjusted life-year; ICER: incremental cost-effectiveness ratio

### Sensitivity analysis

**Fig 2:**
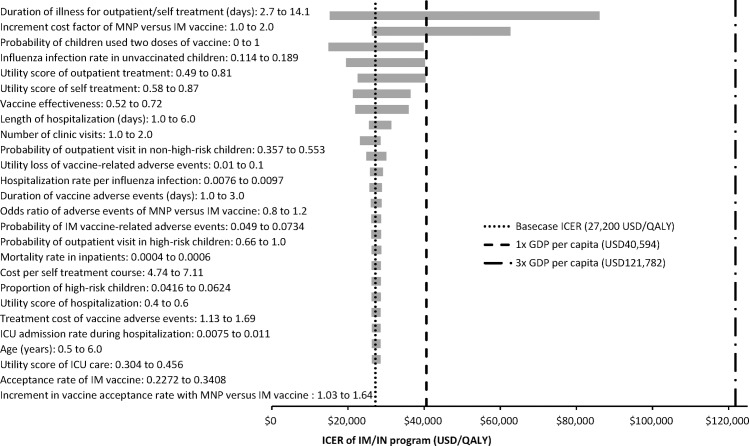
Tornado diagram of ICER of IM/MNP program versus IM program. ICER: incremental cost-effectiveness ratio; IM: intramuscular; MNP: microneedle patch; ICU: intensive care unit; GDP: gross domestic product.

The tornado diagram (Fig 2) showed the impact of one-way variation of all model inputs on the ICER of IM/MNP program. Whilst the ICER remained less than 3x GDP per capita (USD121,782) throughout variation of all model inputs in one-way sensitivity analysis, variation of two model inputs resulted in ICER exceeding 1x GDP per capita (USD40,594). When the duration of illness for outpatient/self-treatment of influenza was less than 5.7 days, or the cost per MNP vaccine administered was >1.39-fold of IM vaccine cost, the ICER of IM/MNP exceeded 1x GDP per capital (USD40,594).

**Fig 3:**
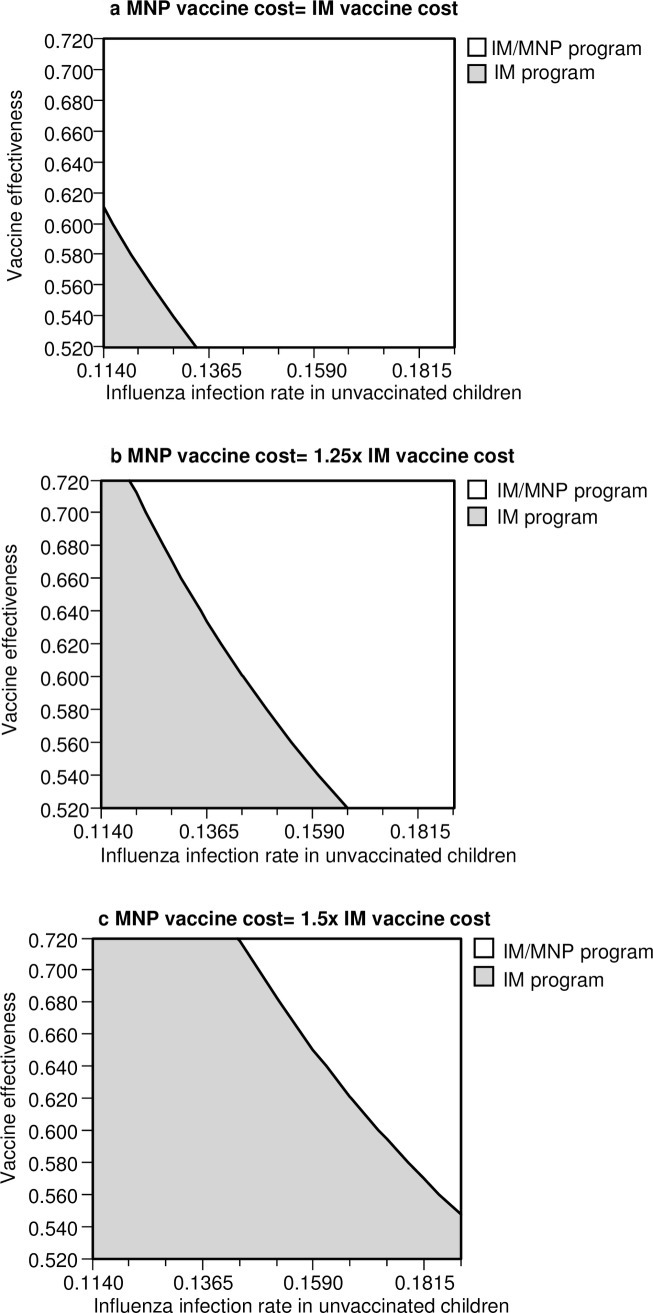
Three-way sensitivity analysis of influenza infection rate in unvaccinated children versus vaccine effectiveness on cost-effectiveness of IM/MNP program at three cost levels of MNP (WTP threshold = 1x GDP per capita). IM: intramuscular; MNP: microneedle patch; WTP: willingness-to-pay; GDP: gross domestic product.

Three-way sensitivity analysis of influenza infection rate in unvaccinated children, vaccine effectiveness and three different cost levels of MNP vaccine was shown in Fig 3A–3C. Using 1x GDP per capita (USD40,594) as WTP threshold, the number of variable combinations indicating IM/MNP program to be the preferable option reduced as the MNP vaccine cost increased. At low cost level of MNP vaccine (equaled to IM vaccine cost), the IM/MNP program was the preferred option when the infection rate in unvaccinated children and vaccine effectiveness were higher than 13.4% and 61.3%, respectively. At high cost level of MNP (1.5-fold IM vaccine), the IM/MNP program was the preferred option only when the infection rate in unvaccinated children was higher than 16.4% at the current base-case vaccine effectiveness (63%). When 3x GDP per capita was applied as WTP threshold, all variable combinations at each of 3 cost-levels indicated IM/IN program to be the preferred option.

**Fig 4:**
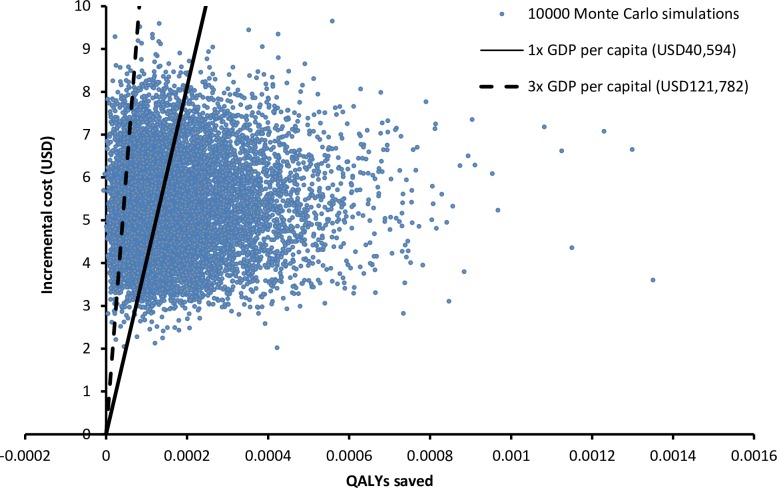
Scatter plot of incremental costs versus incremental QALYs saved by IM/MNP program versus IM program in 10,000 Montel Carlo simulations. IM: intramuscular; MNP: microneedle patch; GDP: gross domestic product; QALY: quality-adjusted life-year.

**Fig 5:**
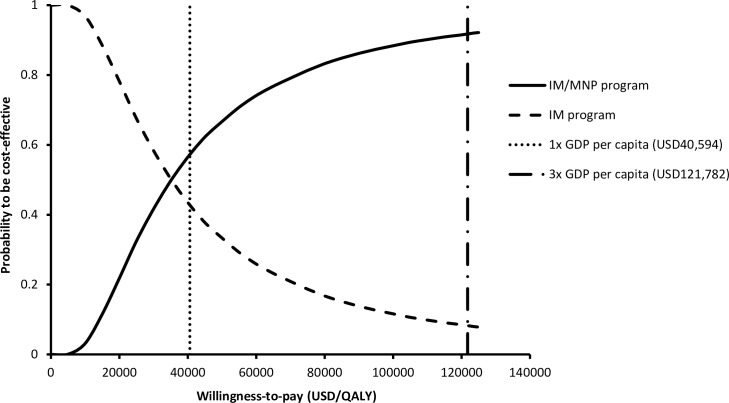
Acceptability curve of each program to be preferred against willingness-to-pay per QALY. IM: intramuscular; MNP: microneedle patch; GDP: gross domestic product; QALY: quality-adjusted life-year.

Probabilistic sensitivity analysis was performed with 10,000 Monte Carlo simulations. Compared to IM program, IM/MNP program was more costly by USD5.36 per child (95%CI: USD5.34–5.38; p<0.001) and more effective by saving 0.000182 QALYs per child (95%CI: 0.000180–0.000186; p<0.001). The scatter plot (Fig 4) of 10,000 simulations showed that the IM/MNP program was more costly than IM program in 100% of simulations and saved QALYs in 99.98% of the time. The probabilities of each program to be cost-effective were examined in the acceptability curve over a wide range of WTP (0–125,000 USD/QALY) (Fig 5). Using 1x GDP per capita as WTP threshold (40,594 USD/QALY saved), IM/MNP program and IM program were the preferred option in 57.28% and 42.72% of the time, respectively. When 3x GDP per capita was used as WTP threshold (121,782 USD/QALY saved), the probability of IM/IN program to be the preferred option increased to 91.68%.

## Discussion

Our study examined the potential cost-effectiveness of offering a needleless option of influenza vaccine (delivered by MNP) to children who had declined IM vaccine from the perspective of Hong Kong's healthcare provider. Base-case analysis showed that the IM/MNP program was more effective than IM program in reducing influenza infection rate, hospitalization rate and influenza-associated mortality rate, with an ICER of USD27,200 per QALY saved. The 10,000 Monte Carlo simulations found the IM/MNP program to be more costly at all time and saved QALYs in over 99.98% of the simulations. The results of present analysis suggested that a modest increase in vaccine coverage provided by an option of MNP to those who declined IM vaccine would result in QALY saving comparing to a program offering IM vaccine alone.

Sensitivity analysis found the acceptance of IM/MNP program as the cost-effective option (preferred option) was subject to the selected WTP threshold. At WTP threshold of 3x GDP per capita (USD 121,782), IM/MNP as the preferred program was highly robust in one-way, three-way and probabilistic sensitivity analysis, with no identified threshold value for all model inputs. At lower WTP threshold (1x GDP per capita = USD 40,594), the robustness of the base-case results was influenced by two model inputs at one-way sensitivity analysis. One of the influential factors (duration of illness of outpatient care and self-treatment) was associated with the QALY loss at outpatient and self-treated cases. The hospitalization rate per influenza case (0.0084) in children population [3] was relative low comparing to elderly population (0.0421) [28], and majority of infected cases were managed in outpatient or self-treatment setting. The influenza-related QALY loss was therefore highly influenced by the duration of illness at the outpatient settings. Another factor identified by one-way sensitivity was the incremental cost factor of MNP versus IM vaccine, and it was similar to the findings of prior cost-effectiveness analysis of influenza vaccination [10,19,20] that the cost of vaccine is highly influential on the cost-effectiveness of influenza vaccination program in both Hong Kong and in the US.

Comparing with injectable influenza vaccine, the needleless formulation (by intranasal route) was reported to achieve higher vaccine acceptance (19.3% versus 12.2%) in a cluster randomized trial evaluating the uptake of influenza vaccine in ten elementary schools with 2,800 students in Canada [29]. Unfortunately, the vaccine effectiveness of intranasal live attenuated influenza vaccine (IN LAIV) among children was significantly lower than the IM inactivated influenza vaccine for two consecutive seasons (2014–2015 and 2015–2016), and the use of intranasal vaccine for 2016–2017 was recently voted down by the CDC’s Advisory Committee on Immunization Practice due to lack of measurable protective benefit (vaccine effectiveness 3%; 95%CI −49% to 37%) during 2015–2016 season [15]. As IN LAIV (the only needleless influenza vaccine) for children is no longer recommended, the vaccine coverage rate by IM vaccine in coming seasons is likely to decline and potentially reduce the clinical benefits of influenza vaccine program for children. The development of MNP for delivery of inactivated influenza vaccine is a highly anticipated technique. The breakthrough development in microfabrication technology enables the production of microneedles, and realizes painless vaccine delivery and elimination of the risks and fears of needle injection. The advantages of MNP include simplification of administration (by minimally trained workers), storage, distribution, and disposal compared with conventional vaccines. The microneedle technology allows expanding access to vaccines for children, even in remote areas, against influenza and other vaccine-preventable diseases including polio, rotavirus, rubella, and tuberculosis. CDC names this technology the potential “game changer” of global vaccination [30].

The present findings, in combination with vaccine coverage, influenza infection rate and vaccine effectiveness data through influenza surveillance, provide insights for healthcare policy makers and providers to consider the potential cost-effective implementation of an upcoming technology for needleless delivery of influenza vaccine in the current children influenza vaccination program. The influenza surveillance in Hong Kong suggested that influenza activity has been increasing continuously over the last three years [1]. As demonstrated by the three-way sensitivity analysis, the likelihood of accepting the IM/MNP program (based upon WTP threshold = 1x GDP per capita) increased with higher baseline influenza infection rate. Despite the less severe manifestations of influenza in children (short length of illness and low hospitalization rate), children play a major role in the dissemination of influenza in households [31,32]. The implementation of IM/MNP program is anticipated to improve vaccine coverage and reduce influenza infection rate in children. It would also potentially decrease secondary transmission of influenza in households with children, and reduce the influenza-related health care utilization in both public and private settings in Hong Kong. The model used in this study contained key economic and clinical inputs that could be readily generalized to other countries by adopting regionally specific model inputs.

The present analysis was limited by the uncertainty of model inputs as well as simplification of real-life events. All inputs of the present model were therefore examined over a wide range of values in the sensitivity analysis to examine their influence on the robustness of model results at different thresholds of WTP. Enhanced protection by herd immunity resulting from increased vaccine coverage was not included in the present model. The model results might therefore understate the beneficial outcomes of a vaccination program with improved coverage. The possibility of Guillain-Barré syndrome was excluded in the model due to the extreme low incidence [33]. The cost-analysis of our study was conducted from the perspective of healthcare provider (instead of societal perspective) and only direct medical costs were included. The indirect cost (loss of productivity) of caregivers to care for infected children in outpatient setting (majority of pediatric cases of influenza) was not included and the economic benefits of the IM/MNP program might not be fully assessed. We only accounted for the direct costs of administered vaccines but not refused or wasted doses, and a budget analysis is warranted to estimate the operational cost of an IM/MNP program.

In conclusion, an influenza vaccination program that offered a needleless MNP for inactivated influenza vaccine as an alternative to children who declined IM vaccine appeared to reduce infection rate, hospitalization rate and mortality and save QALY at higher cost when compared to a program only offered IM vaccine. The acceptance of the IM/MNP program as the preferred program was subject to the WTP threshold, duration of illness in outpatient settings for infected children, and cost of MNP vaccine relative to IM vaccine.
